# The small blood vessels in areas of lymphocytic infiltration around malignant neoplasms.

**DOI:** 10.1038/bjc.1982.194

**Published:** 1982-08

**Authors:** A. J. Freemont

## Abstract

**Images:**


*Br. J. Cancer* (1982) **46,** 283

***Short Communication***

### THE SMALL BLOOD VESSELS IN AREAS OF LYMPHOCYTIC INFILTRATION AROUND MALIGNANT NEOPLASMS

A. J. FREEMONT

*From the Department of Pathology University of Manchester Medical School, Manchester M13 9PT*

Received 1 March 1982 Accepted 2 April 1982

LYMPHOCYTIC INFILTRATES have been described in association with many different human non-lymphoid malignant neoplasms (Underwood, 1974) and in a few, such as medullary carcinoma of the breast, are characteristic of the tumour. Many investigators have reported an association between improved prognosis and lymphocyte infiltration (Bloom *et al.*, 1970; Hawley *et al.*, 1970; Kiely *et al.*, 1972; Lauder & Aherne, 1972). Others have not found such a favourable association (White, 1927).

The lymphocytic infiltrate is often dense, particularly around the periphery of the tumour. The factors responsible for cellular accumulation around neoplasms are poorly understood (Edwards *et al.*, 1973; Wasserman *et al.*, 1974). Studies of lymphocyte migration into all lymphoid tissues (except the spleen) and sites of chronic inflammation in experimental animals (Graham & Shannon, 1972) have shown that most lymphocytes pass from blood to tissue through post-capillary vessels, specialized for increased lymphocyte traffic (Gowans & Knight, 1964) known as high endothelial venules (HEV). They have distinctive structural and histochemical features (Smith & Henon, 1959; Anderson *et al.*, 1976) believed to reflect their functional specialization.

In this study, the structure and histochemistry of vessels within tumour lymphocytic infiltrates have been compared with the HEV of lymph nodes, in order to ascertain whether they share features not found in vessels elsewhere.

Thirty-six primary carcinomas of 11 different types with lymphocytic infiltrates (squamous-cell carcinoma of larynx (1), oesophagus (2) and lung (3), adenocarcinoma of stomach (3), rectum (4) and prostate (4), medullary (3) and infiltrating duct (5), carcinoma of breast, seminoma (4), basal-cell carcinoma of skin (4) and transitional-cell carcinoma of bladder (3) 11 examples of 5 similar carcinomas without infiltrates (adenocarcinoma of stomach (1) and prostate (2), infiltrating-duct carcinoma of breast (3), squamous-cell carcinoma of oesophagus (2) and transitional-cell carcinoma of bladder (3)) and specimens from 3 normal lymph nodes, were processed for light microscopy, histochemistry and electron microscopy.

For light microscopy 4 $\mu$m sections of formalin-fixed paraplast-embedded tissue were stained with haematoxylin and eosin, methyl green pyronin and periodic acid–Schiff (PAS). Sections were also stained with Azure A using the method described by Ball & Jackson (1953) with and without ribonuclease treatment at a 1:1000 dilution in phosphate buffer (pH 6) for 3 h at 37°C.

Non-specific-esterase (NSE) activity was demonstrated on tissue fixed for 18 h in ice-cold formol-sucrose using $\alpha$-naphthyl propionate in a simultaneous

FIG. 1.—A plump endothelium-lined vessel (H) with lymphocytes in its lumen and wall, in the lymphocytic infiltrate (L) around an adenocarcinoma of the stomach (T). H. & E. ×400.

coupling azo-dye technique (Freemont & Davies, 1982). Substrate-free negative controls were also prepared.

Specimens for electron microscopy, fixed for 4 h in cacodylate-buffered 2·5% gluteraldehyde (pH 7·4) were washed in 0·1M cacodylate buffer and diced to 1mm cubes. They were post-fixed in 1% osmium tetroxide in phosphate buffer (pH 7·4) at 4°C for 1½ h, dehydrated in graded alcohols and propylene oxide and embedded in EMIX resin. Sections (0·5 μm) were stained with 1% toluidine blue in borax, and suitable areas selected for ultrathin sectioning. Grids were double stained with uranyl acetate and Reynolds' lead citrate and examined in a Philips 301 electron microscope.

The lymphocytic infiltrate was densest around the periphery of the neoplastic cell mass and in the surrounding connective tissue. In no specimen was all the surface covered with lymphocytes, and in some, such as prostatic adenocarcinoma, lymphocytes and tumour were infrequently in contact. Lymphocytes were also found within the tumour mass, most commonly in fibrous septae, but occasionally amongst groups of malignant cells. Within the areas of densest lymphocyte aggregation was a population of blood vessels which were morphologically and cytochemically distinct from vessels elsewhere. Throughout the tissue examined, most of the vessels were lined by a flattened endothelium with uniformly staining spindle-shaped nuclei and scanty cytoplasm. The vessels within the lymphocytic infiltrate, in contrast, contained plump endothelial cells with large ovoid open or reticulated nuclei and abundant cytoplasm, which gave the cells a cuboidal appearance (Fig. 1). Unlike that of the flattened endothelium, the cytoplasm of

FIG. 2.—NSE+ blood vessel (H) in the lymphocytic infiltrate (L) about an infiltrating-duct carcinoma of breast (T) which is also positive. ×200.

these cells was pyroninophilic, exhibited ribonuclease-labile metachromasia with Azure A and numerous dense NSE+ granules (Fig. 2). The vascular basement membrane was thicker than in other vessels and stained strongly with PAS. The most striking feature of these vessels was the large number of lymphocytes within the basement membrane and around the vessel, where they often appeared to be arranged in concentric circles. Lymphocytes were not found in such intimate relationship with endothelial cells in other vessels, nor were plump-endothelium-lined vessels found in those tumours without a lymphocytic infiltrate. These features are identical to those described as peculiar to the lymph-node HEV of rodents. Human lymph-node HEV have not previously been investigated in such detail, but in the 3 lymph nodes in this study the same nuclear and cytoplasmic characteristics were recorded.

The plump endothelium, both in the tumour lymphocytic infiltrate and the lymph-node HEV, exhibited a similar ultrastructure to that described for the HEV of rats and mice (van Deurs & Ropke, 1974; Anderson *et al.*, 1976). Whilst the amount of cytoplasm varied from cell to cell, in most it accounted for more than half the cell volume and bulged into the vascular lumen. The luminal pole of the cell was particularly rich in organelles. The cytoplasmic pyroninophilia and metachromasia could be explained by the many free single and clustered ribosomes.

Rough endoplasmic reticulum and Golgi cisternae and vesicles, whilst present throughout the cytoplasm, were most prominent near the lumen and closely associated with numerous mitochondria,

FIG. 3.—A high endothelial type vessel (HEV) in the lymphocytic infiltrate surrounding a medullary carcinoma of the breast, showing the nuclear morphology and abundant cytoplasm of the endothelial cells (H) and a lymphocyte (L) in transit across the wall. × 1000.

secondary lysosomes and multivesicular bodies, containing up to 20 vesicles in a dense matrix. Microfilaments were present toward the surfaces of the cell and occasional tight junctions were seen. The thickened $PAS^+$ basement membrane was shown to be a composite zone consisting of vascular basement membrane, reticulin fibres and pericyte lattice.

Lymphocytes within the vessel walls were situated in the inter-endothelial spaces, between the endothelial cells and the basement membrane, and within the layers of the basement membrane and pericyte lattice (Fig. 4). In the latter, the lymphocytes were flattened circumferentially about the vessel. By contrast, the flattened endothelium had much less cytoplasm and far fewer organelles; particularly Golgi material, free ribosomes, dense bodies and rough endoplasmic reticulum. No lymphocytes were seen in the walls of vessels lined by these cells.

The evidence points to there being a population of blood vessels within the lymphocytic infiltrate around malignant neoplasms, which exhibit those structural and metabolic features of lymph-node HEV which are believed to reflect their specialized function. Furthermore they are not seen outside areas of heavy lymphocyte concentration.

Since their original description (Thomé, 1898) lymphoid tissue HEV have been regarded as specialized microvascular structures. The occurrence of lymphocytes within their walls led investigators to conclude that these vessels were important in the transfer of cells from blood to tissue (Schumacher, 1899; Hummel, 1935). This view was confirmed when recirculating lymphocytes were shown to

FIG. 4.—A plump endothelium-lined vessel showing the lumen (LU) and 3 lymphocytes (L) between the cell (H) and complex basement membrane (B) in a seminoma. ×6000.

selectively migrate from blood vessels to lymph nodes at this site (Gowans & Knight, 1964).

The similarities between the vessels within the tumour lymphocytic infiltrate and lymph-node HEV, particularly their intimate association with lymphocytes and their absence from areas other than those of heavy lymphocyte aggregation, may well indicate a similar specialized function. Whilst the evidence for increased lymphocyte traffic into the infiltrate through the walls of these vessels is purely circumstantial, it is conceivable that they represent the route by which a significant number of lymphocytes enter the area around the tumour.

If, as has been proposed, a lymphocytic infiltrate around a neoplasm improves the prognosis, and these vessels represent the mechanism by which increased lymphocyte traffic into the tumour is achieved, their presence will beneficially influence the course of malignant disease. Under these circumstances, the factors controlling the development of the vessels and the mechanisms by which they initiate and regulate lymphocyte traffic assume considerable importance.

Unfortunately no useful parallel can be drawn from the development of lymph-node HEV. It has been shown that the plumpness of the endothelial cells is related to lymphocyte traffic into the tissue (Hummel, 1935). But the initial stimulus to their formation is unknown. Equally little is known about the control of lymphocyte migration by the endothelial cells. Andrews *et al.* (1980) autoradiographically the production of a sulphated glycoconjugate by these cells, which they believe may be functionally related to the transport of lymphocytes from the blood to the lymph nodes. The plump endothelial cells of the HEV-like vessels, in the infiltrate about malignant tumours, have the organelles shown to be involved in the synthesis of the sulphated material, but its production has not been demonstrated.

Whilst lymphocyte infiltration is an important reaction to a carcinoma, the mechanism of cellular migration is poorly understood. Vascular specialization could be central to the control of lymphocyte traffic into the tumour, as it is elsewhere, and further investigation is needed to improve our understanding of its place in this response to malignant disease.

This work was supported by a grant from Manchester Central District Grants Committee.

### REFERENCES

ANDERSON, N. D., ANDERSON, A. O. & WYLIE, R. G. (1976) Specialised structure and metabolic activities of high endothelial venules in rat lymphatic tissue. *Immunology*, **31**, 455.

ANDREWS, P., FORD, W. L. & STODDART, R. W. (1980) Metabolic studies of high-walled endothelium of post-capillary venules in rat lymph nodes. *Blood Cells and Vessel Walls: Functional Interactions. Ciba Foundn Symp.*, **71**, p. 211.

BALL, J. & JACKSON, D. S. (1953) Histological, chromatographic and spectrophotometric studies of toluidine blue. *Stain Tech*, **28**, 33.

BLOOM, J. H. G., RICHARDSON, W. W. & FIELD, J. R. (1970) Host resistance and survival in carcinoma of breast: A study of 104 cases of medullary carcinoma of breast carcinoma followed for 20 years. *Br. Med. J.*, i, 181.

EDWARDS, S. J., ROWLAND, G. F. & LEE, M. R. (1973) Reduction of lymphocyte transformation by a factor produced by gastrointestinal cancer. *Lancet*, i, 687.

FREEMONT, A. J. & DAVIES, J. S. (1982) Acid esterase activity in lymphocytes and other cells: A comparison of six 4-naphthyl-based substrates. *Med. Lab. Sci.* (In press.)

GOWANS, J. L. & KNIGHT, E. J. (1964) The route of recirculation of lymphocytes in the rat. *Proc. R. Soc. Lond.* [Biol.], **159**, 257.

GRAHAM, R. C. & SHANNON, S. L. (1972) Peroxidase arthritis. II. Lymphoid cell–endothelial interactions during a developing immunologic inflammatory response. *Am. J. Pathol.*, **69**, 7.

HAWLEY, P. R., WESTERHOLM, P. & MORSON, B. C. (1970) Pathology and prognosis in carcinoma of the stomach. *Br. J. Surg.*, **57**, 877.

HUMMEL, K. P. (1935) The structure and development of the lymphatic tissue in the intestine of the albino rat. *Am. J. Anat.* **57**, 351.

KIELY, E., GREALLY, M. & GREALLY, J. (1972) On the significance of lymphoid cell infiltration in hypernephromas. *Ir. J. Med. Sci.*, **141**, 108.

LAUDER, I. & AHERNE, W. (1972) The significance of lymphocytic infiltration in neuroblastoma. *Br. J. Cancer.*, **26**, 321.

SCHUMACHER, S. VAN (1899) Ueber Phagocytose und die Abfuhrwege der Leucocyten in den Lympdrusen. *Arch. Mikrosk. Anat.*, **54**, 311.

SMITH, C. & HENON, B. K. (1959) Histological and histochemical study of high endothelium of post-capillary veins of the lymph node. *Anat. Rec.* **135**, 207

THOMÉ, R. (1898) Endothelien als Phagocyten (aus den Lymphdrusen von *Macacus cynomalgus*). *Arch. Mikrosk Anat.*, **52**, 820.

UNDERWOOD, J. C. E. (1974) Lymphoreticular infiltration in human tumours: Prognostic and biological implications: A review. *Br. J. Cancer*, **30**, 538.

VAN DEURS, B. & ROPKE, C. (1974) The postnatal development of high-endothelial venules in lymph nodes of mice. *Anat. Rec.* **181**, 659.

WASSERMAN, S. I., GOETZL, E. J., ELLMAN, L. & AUSTEN, F. K. (1974) Tumour-associated eosinophilotactic factor. *N. Engl. J. Med.*, **290**, 420.

WHITE, W. C. (1927) Late results of operation for carcinoma of the breast. *Ann. Surg.* **86**, 695.


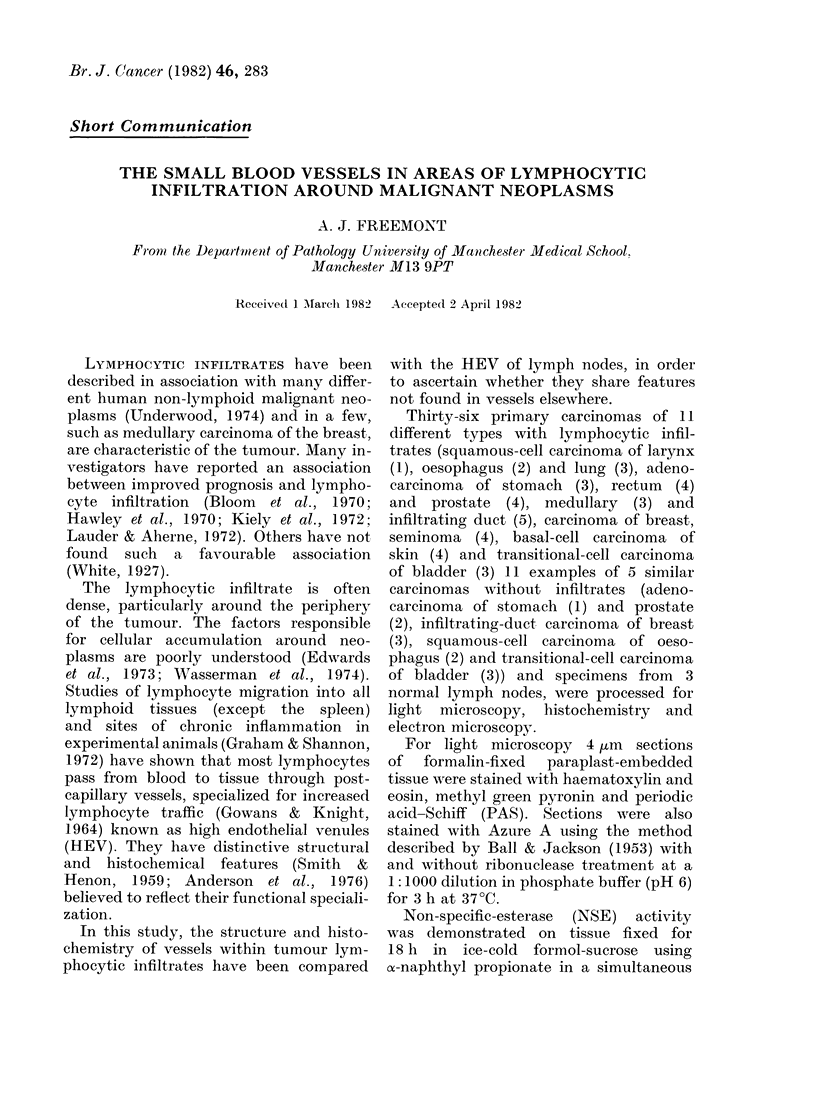

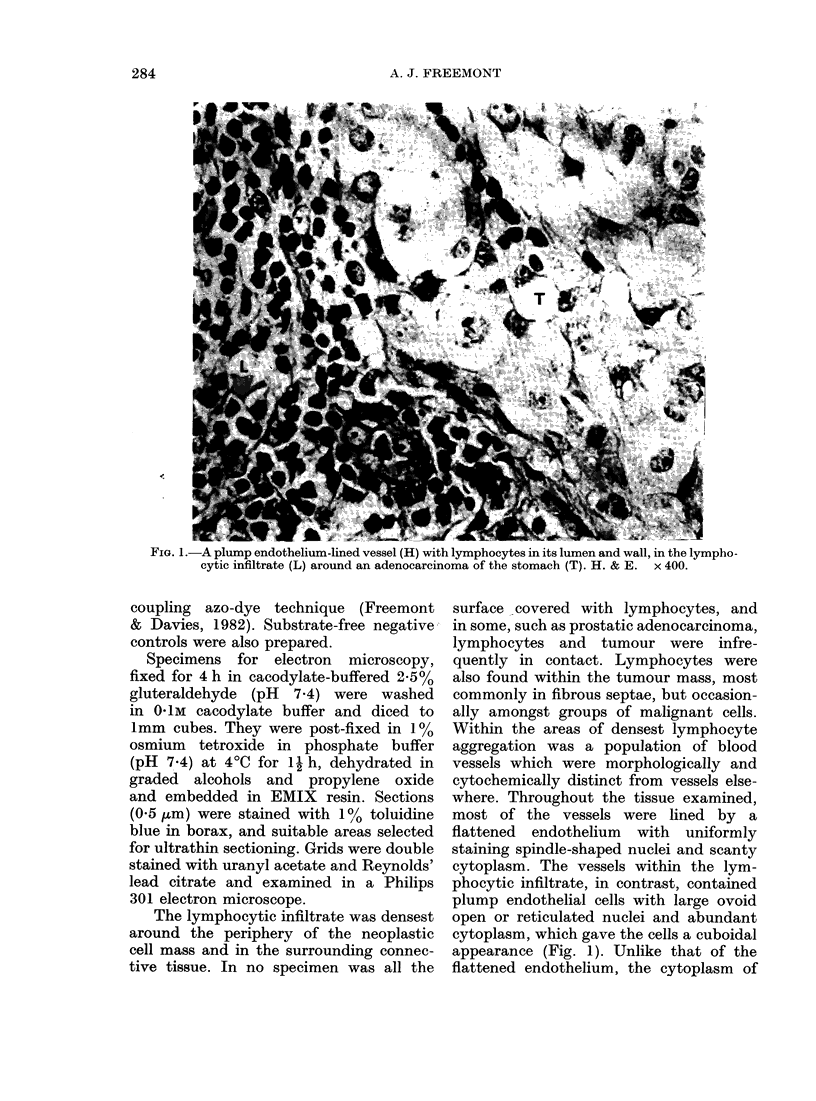

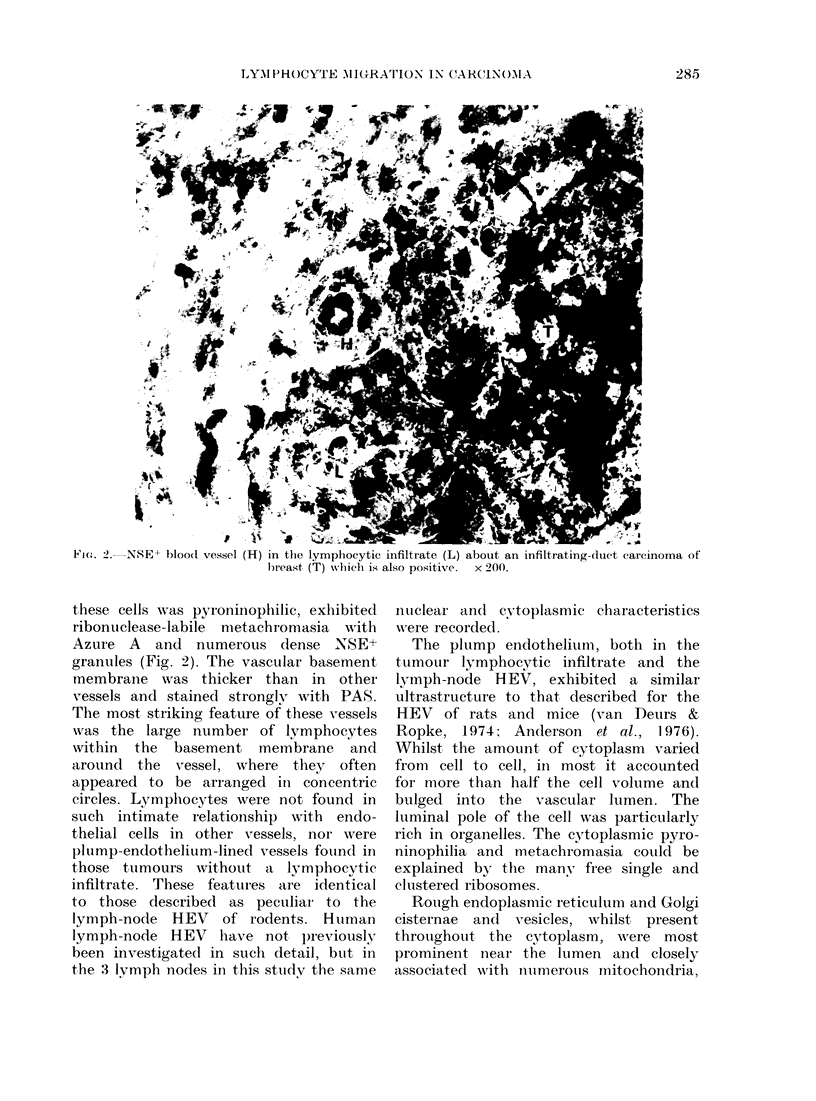

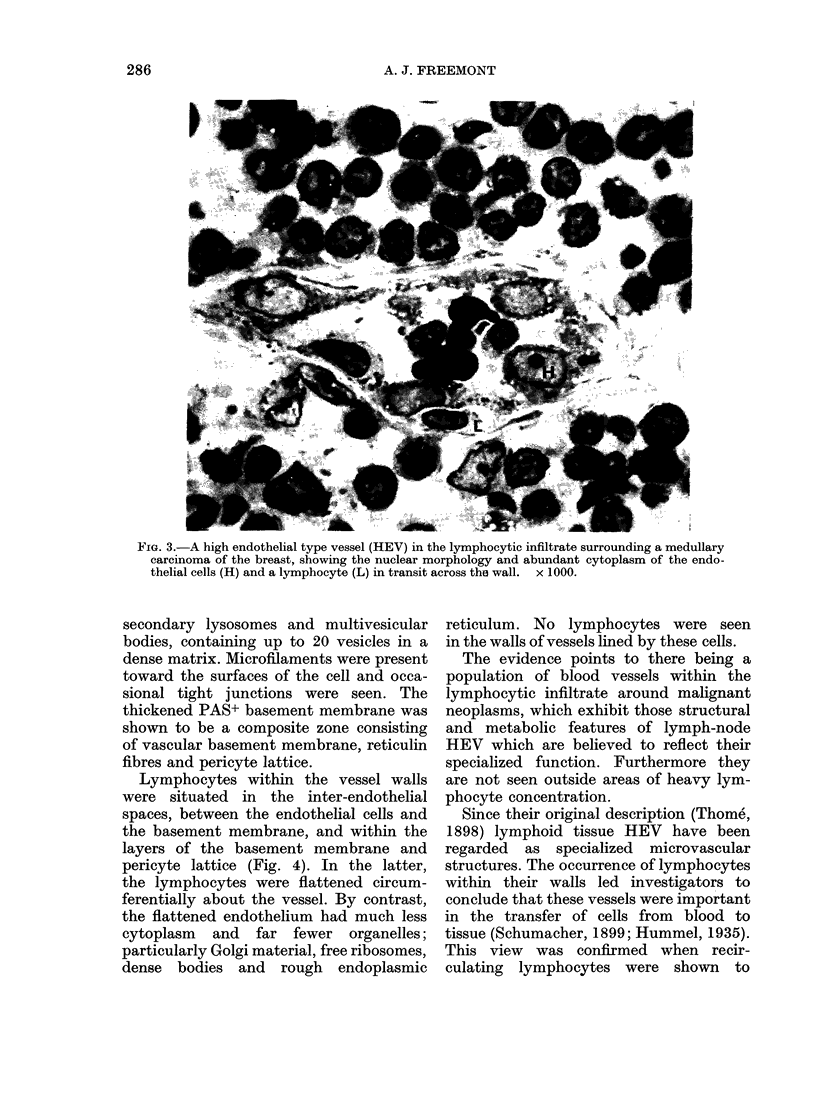

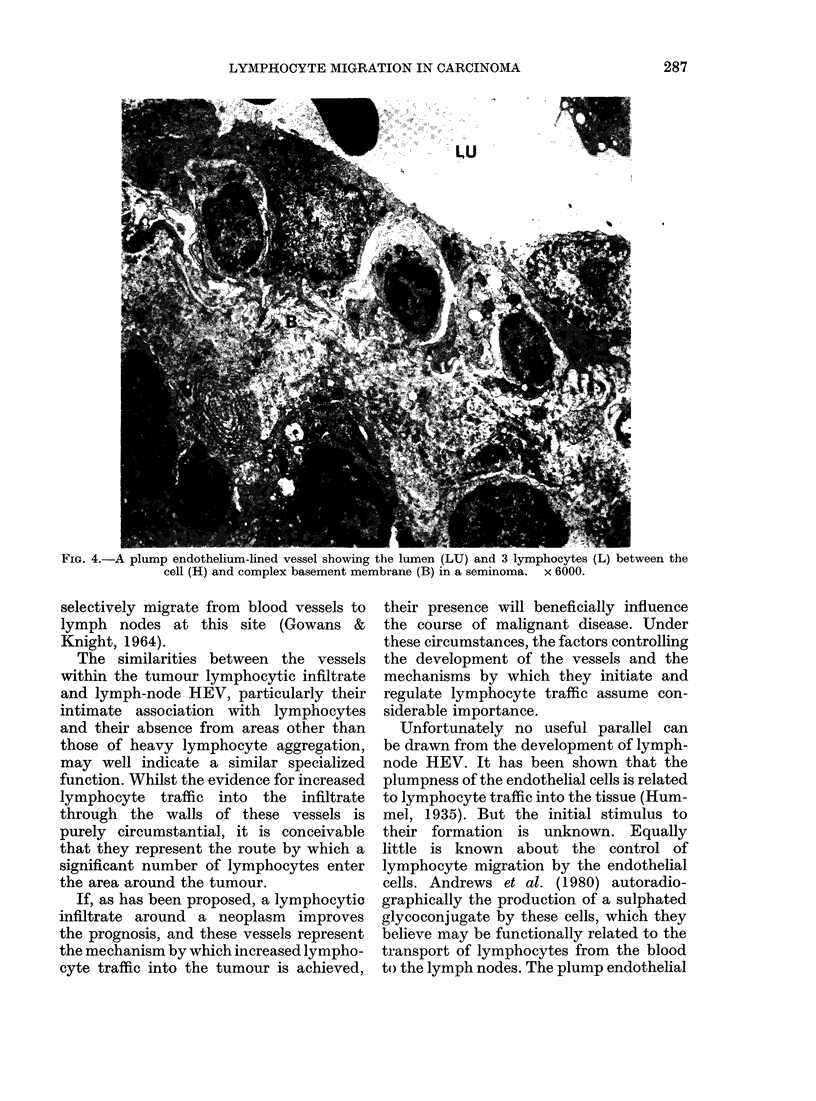

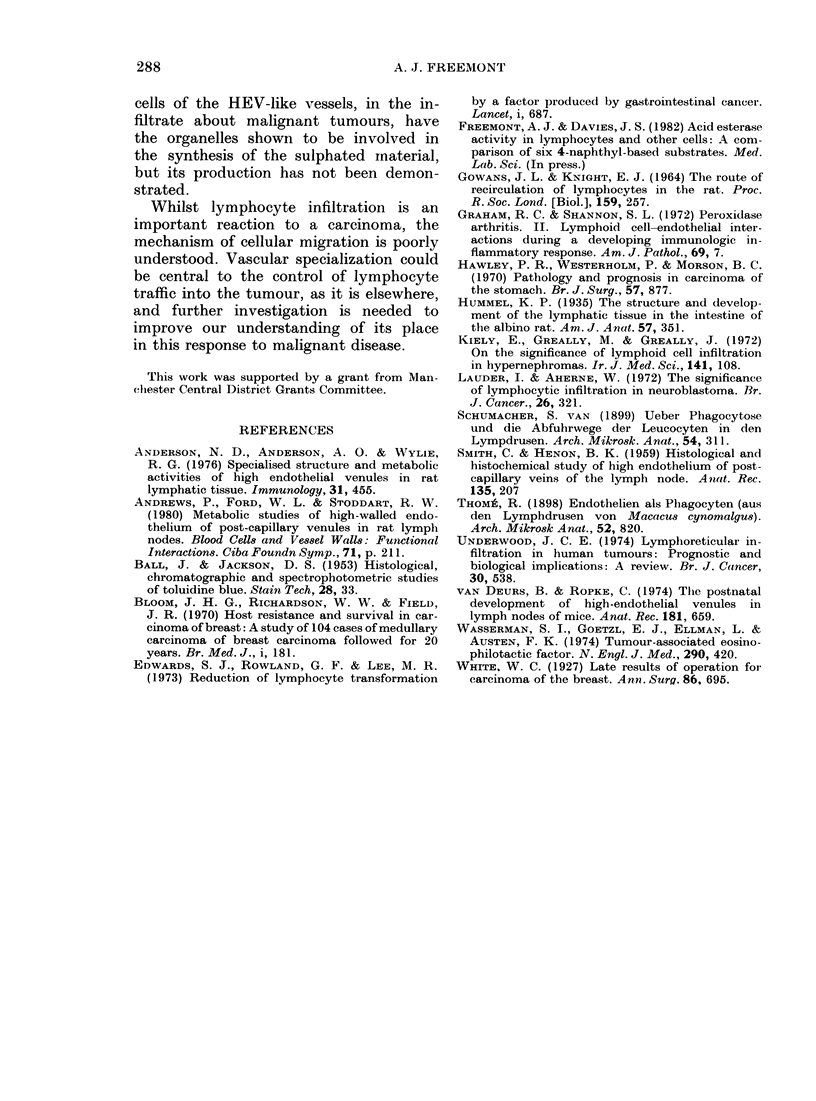


## References

[OCR_00350] Anderson N. D., Anderson A. O., Wyllie R. G. (1976). Specialized structure and metabolic activities of high endothelial venules in rat lymphatic tissues.. Immunology.

[OCR_00363] BALL J., JACKSON D. S. (1953). Histological, chromatographic and spectrophotometric studies of toluidine blue.. Stain Technol.

[OCR_00368] Bloom H. J., Richardson W. W., Field J. R. (1970). Host resistance and survival in carcinoma of breast: a study of 104 cases of medullary carcinoma in a series of 1,411 cases of breast cancer followed for 20 years.. Br Med J.

[OCR_00375] Edwards A. J., Rowland G. F., Lee M. R. (1973). Reduction of lymphocyte transformation by a factor produced by gastrointestinal cancer.. Lancet.

[OCR_00388] GOWANS J. L., KNIGHT E. J. (1964). THE ROUTE OF RE-CIRCULATION OF LYMPHOCYTES IN THE RAT.. Proc R Soc Lond B Biol Sci.

[OCR_00393] Graham R. C., Shannon S. L. (1972). Peroxidase arthritis. II. Lymphoid cell-endothelial interactions during a developing immunologic inflammatory response.. Am J Pathol.

[OCR_00399] Hawley P. R., Westerholm P., Morson B. C. (1970). Pathology and prognosis of carcinoma of the stomach.. Br J Surg.

[OCR_00409] Kiely E., Greally M., Greally J. (1972). On the significance of lymphoid cell infiltration in hypernephromas.. Ir J Med Sci.

[OCR_00414] Lauder I., Aherne W. (1972). The significance of lymphocytic infiltration in neuroblastoma.. Br J Cancer.

[OCR_00424] SMITH C., HENON B. K. (1959). Histological and histochemical study of high endothelium of post-capillary veins of the lymph node.. Anat Rec.

[OCR_00435] Underwood J. C. (1974). Lymphoreticular infiltration in human tumours: prognostic and biological implications: a review.. Br J Cancer.

[OCR_00446] Wasserman S. I., Goetzl E. J., Ellman L., Austen K. F. (1974). Tumor-associated eosinophilotactic factor.. N Engl J Med.

[OCR_00451] White W. C. (1927). LATE RESULTS OF OPERATION FOR CARCINOMA OF THE BREAST.. Ann Surg.

[OCR_00441] van Deurs B., Röpke C. (1975). The postanatal development of high-endothelial venules in lymph nodes of mice.. Anat Rec.

